# Anthropometric indices, blood pressure, and lipid profile status among women using progestin-only contraceptives: comparative cross-sectional study

**DOI:** 10.1186/s12905-021-01178-8

**Published:** 2021-01-23

**Authors:** Mulu Shiferaw, Woldeteklehaymanot Kassahun, Belay Zawdie

**Affiliations:** 1 Department of Nursing, College of Health Science, Woldia University, 400, Woldia, Ethiopia; 2 Department of Medical Laboratory Science, College of Medicine and Health Sciences, Arbaminch University, Arba Minch, Ethiopia; 3 Department of Biomedical Sciences, Faculty of Medical Sciences, Institute of Health, Jimma University, Jimma, Ethiopia

**Keywords:** Anthropometric indices, Blood pressure, Progestin-only contraceptive, Lipid profiles, Ethiopia

## Abstract

**Background:**

The major types of hormonal contraception used currently in Ethiopia are progestogen-only. This study aimed to assess the differences in anthropometry indices, blood pressure, and lipid profile among women using progestin-only contraceptives in selected health facilities of Jimma town, southwest Ethiopia.

**Methods:**

A facility-based comparative cross-sectional study was conducted among women of reproductive age using Depo-medroxyprogesterone acetate (DMPA) and implant in selected health facilities from April 11 to May 11, 2019. A total of 146 women (45 DMPA and 51 implant users and 50 non-users) were selected randomly for inclusion in the study. One-way analysis of variance was used to examine variations in health outcomes while Bonferroni post-hoc tests were conducted to determine significance of variations between multiple outcomes.

**Results:**

There was a statistically significant difference in the mean Body Mass Index (*p* = 0.045), Hip-to-waist ratio (*p* = 0.012), systolic blood pressure (*p* = 0.027), diastolic blood pressure (DBP) (*p* = 0.017), total cholesterol (TC) (*p* = 0.005), low-density lipoprotein (*p* = 0.023) and triacylglycerol (TAG) (*p* = 0.000) between women using progestin-only contraceptives and non-users. DMPA users had higher TC (*p* = 0.024) than non-users. Results from Pearson correlation analysis showed that DBP of DMPA users was higher with longer duration of use.

**Conclusion:**

The findings suggest the need for family planning service providers to undertake appropriate client profiling before recommending a particular method to women seeking the services in order to minimize adverse health risks, especially for those who may have pre-existing conditions.

## Background

The major types of hormonal contraception used currently in Ethiopia are progestogen-only. They are available in the form of progestogen-only pills, subdermal implants, injection and hormones-releasing intrauterine systems [1]. The chemical composition and type of the progestin-only contraceptives used by women in this study were Depo-Medroxyprogesterone acetate (DMPA) with a dose of 150 mg/ml Medroxyprogesterone acetate which is injectable and implant (Implanon- 68 mg of levonorgestrel and Jadelle implants, a set of two flexible cylindrical implants, each containing 75 mg of the progestin levonorgestrel) which is implanted under the arm.

The mechanism of action of progestogen-only contraceptives is by affecting cervical mucus, making it hostile to ascending sperm and by thinning and atrophying the endometrium, thereby preventing sperm transport and implantation [2]. Higher dose progestogen-only methods will also act centrally and inhibit ovulation [3]. Obesity, nausea, breakthrough bleeding, breast tenderness, increased blood pressure and high level of cholesterol are commonly reported progestin-only contraceptive side effects [4]. Progestin-only contraceptives have been shown to alter the lipid profile, body weight, and blood pressure among various population groups with different patterns of dyslipidemia and cardiovascular (CV) disease risks [5].

Most of the side effects of progestins on lipid metabolism, such as reducing high-density lipoprotein (HDL), are thought to be because of the androgen receptor activation [6]. The decrement in HDL levels due to progesterone administration contributes to increments in the expression of Scavenger Receptor B, class 1 (SR-B1) in the liver, and increases in plasma hepatic lipase activity, but has a minor effect on lipoprotein lipase [5]. A rise in SR-B1 enhances the transfer of cholesterol from HDL particles into the hepatocyte and a decrease of plasma HDL cholesterol levels [7]. An increase in hepatic lipase activity also leads to the hydrolysis of triacylglycerol (TG) and phospholipase on HDL [7]. This reaction results in the formation of smaller HDL particles, the release of APO A-I, and increased APO A-I degradation [8]. The mechanism of how progesterone administration might affect low-density lipoprotein (LDL) levels is not well understood. It is reported that progesterone can antagonize the ability of estrogen to stimulate LDL receptor expression in the liver, which could lead to a decrease in hepatic LDL receptors and an increase in plasma LDL cholesterol levels [9].

Although several investigations have been conducted on the relationship between DMPA and lipid profiles, results are not consistent among various studies. One study demonstrated that DMPA does not affect serum lipids [10] while others have shown an adverse relationship [11] and beneficial effects of DMPA [12]. Similarly, there is a controversy about the effect of implant on lipid profile. One study found that it was a reducing agent while another found it to be an increasing agent of lipid profile [13, 14].

Weight gain is also associated with hormonal contraceptive utilization. However, this association remains controversial. One study found that hormonal contraceptives (HCs) have no effect on weight gain [15], but other studies found adverse effects [16, 17]. Similarly, blood pressure among DMPA users is controversial [18, 19]. A systematic review concluded that studies on the association between hormonal contraceptives and weight gain have limitations and that the nature of association, if any, remains to be clarified [20]. The aim of this study was therefore to assess the differences in anthropometric indices, blood pressure, and lipid profile status among women using progestin-only contraceptives.

## Methods

### Study setting

This was a facility-based comparative cross-sectional study conducted from April 11 to May 11, 2019, in purposely selected health facilities in Jimma Town, Jimma zone, Oromia Regional State, southwest Ethiopia. The health facilities were Shenen Gibe General Hospital, Jimma Health Center, and Marie-Stopes International Clinic- Jimma, with a flow of 3136, 2328 and 2880 clients per year, respectively.

### Eligibility criteria

#### Inclusion criteria

Women of reproductive age (15–49 years) who had been using DMPA for at least three months or implant for at least one month were targeted for inclusion in the study.

#### Exclusion criteria

Women of reproductive age using the methods who had a history of chronic illness including diabetes, hypertension, liver dysfunction, cardiovascular disorders (CVD) and lipid profile and blood pressure (BP) affecting drug (including corticosteroids, antipsychotics, diuretics, anticonvulsants, retinoid, and antiviral) and breastfeeding mothers were excluded by reviewing their medical records and/or by asking. Women with suspected pregnancy were checked through rapid Human chorionic gonadotropin (HCG) test and those positive for HCG excluded from the study. Additionally, women with a family history of diabetes and hypertension, and women having physical deformity were excluded from the study.

### Sample size determination

The sample size was determined by using G*power software version 3.1 for mean difference between two independent groups assuming a 5% level of significance (α) and 80% power (β) with an effect size greater than 0.5.

The mean and standard deviation of total cholesterol (TC) were taken from a study done in Addis Ababa [11], to calculate the minimum sample size for DMPA users. The procedure yielded a minimum sample size of 45 women, which was then proportionally allocated to each of the three health facilities as follows: 15 for Jimma Health Center, 7 for Marie Stopes International Clinic-Jimma Ethiopia, and 23 for Shenen Gibe General Hospital.

For implant users, the mean and standard deviation of Body Mass Index (BMI) were taken from a study done in Ghana [21], and the minimum sample size was 51. The sample size was proportionally allocated to each of the three health facilities as follows: 11 for Jimma Health Center, 26 for Marie Stopes International Clinic-Jimma Ethiopia, and 14 for Shenen Gibe General Hospital. A total of 50 women who did not use any of the contraceptives were selected as controls.

### Sampling method

A systematic random sampling method was used to select each study participant by using an interval of 8 and 5 for DMPA and implant users respectively. Participants were recruited from women seeking services at the health facilities. Client flow data for each contraceptive method for the previous month were used to determine the sampling interval. Eligible participants were identified through interviews and review of medical records.

### Data collection and measurement

#### Questionnaire

Behavioral data (including Khat chewing, cigarette smoking, physical exercise, alcohol drinking and dietary habit) were collected using the WHO STEPS Questionnaire (available at https://www.who.int/ncds/surveillance/steps/instrument/en/) adapted to the local context based on the study objectives through face to face interview [22]. The interviews were conducted by trained nurses in a room set aside for the study purpose in each health facility.

#### Anthropometric measurements

The height of the study participants was measured using a stadiometer (Seca, Germany) to the nearest 0.1 cm with the subjects positioned on the Frankfurt Plane. Before starting the measurements, the stadiometer was checked using calibration rods. Weight was measured using a digital glass weight scale to the nearest 0.1 kg with the subjects wearing light closes and shoes taken off. To check the validity of the scale, a known weight was measured every morning and in between the measurements [19]. Waist circumferences were measured at the midway between the lowest costal margin at the midclavicular line and the anterior superior iliac spine using fixed tension tape, while hip circumference was measured at the level of the greater trochanter of the femur with the subjects wearing a pant [23].

All anthropometric measurements were done in triplicate and the average value was used for further analyses. Standardization exercise was done to reduce inter-observer error [19]. BMI was calculated as the weight in kilogram (kg) divided by height in meters squared (kg/m^2^), and hip-to-waist ratio was calculated as the hip circumference in centimeter (cm) divided by waist circumference in cm.

#### Blood pressure measurement

Blood pressure was measured using Omron M2 Basic Blood Pressure Monitor with small, medium, and large cuff size, as appropriate for each participant, 15 min after the clients arrived at the facility. The subsequent measurements were done 5 min apart. Following the WHO recommendation, the mean systolic and diastolic blood pressure was considered for analysis [19].

#### Lipid profiles analysis

Fasting venous blood sample of five milliliter (ml) (after 8 h of fasting) was collected from the medial cubital vein of the left arm with a 19-gauge syringe (adult size) then transferred to a serum separator tube (SST) of 5 ml volume by experienced professional nurses. The blood drawing trial was performed at most two times. If the first trial failed, the unused arm was used for the second. When adverse events such as uncontrolled bleeding occurred, safety precautions were applied with respect to the standard guideline, and patient safety was assured by placing and stopping the bleeding using sterile gauze and banding at the site. Confidentiality of the participants information was kept by using code labels and no name was used to identify samples throughout the study process.

After the blood sample was collected, it was left at room temperature for 30 min until it became completely clotted. It was then kept in 2–8 °C refrigerator for no more than 8 h until prepared for storage. After blood was centrifuged at 3000 RPM for 5 min, the separated serum was stored at -80 °C with Nunc tube until analysis. Lipid profiles were analyzed from serum by Architect c4000 Automated Chemistry analyzer (Abbott, USA) at International Clinical Laboratory according to Standard Operating Procedure (SOP) from serum. Lipid profiles concentrations were reported as mg/dl.

### Data analysis and presentation

Data were entered in Epidata version 4.4.3.1 and exported to IBM SPSS version 25 for analysis. One-way ANOVA was used to compare the mean of lipid profiles, blood pressure, anthropometric indices of DMPA users, implant users, and non-users (control). The Bonferroni post hoc test was used for multiple comparisons. A *p* value of < 0.05 was considered to be statistically significant. Pearson correlation was used to identify the association between outcome variables (anthropometric indices, blood pressure and lipid profiles) and the duration of DMPA and implant use. The normality and homoscedasticity of the data were checked by using the Shapiro–Wilk test and Levene’s test respectively. All values were reported as the means ± standard deviation (SD). The results are presented in charts, tables, and figures.

### Data quality assurance

To ensure quality, the collected data were checked for completeness, accuracy, and clarity by data collection supervisors and the principal investigator. The principal investigator also ensured that source documents were maintained for each participant in the study comprising the investigator’s copy of the signed informed consent, behavioral and medical information, according to applicable good clinical practices (GCP) requirements. Data collectors were trained on how to approach study subjects, sample collection, handling of biological samples, and how to administer the questionnaire. Quality checking was done daily after data collection and amendments made before the next data collection.

All procedures for taking and analyzing biological samples were carried out by professionals. All laboratory procedures were carried out as per the approved protocol/ standard operating procedures (SOPs) of the laboratory.

### Ethical and environmental considerations

Ethical clearance was obtained from Jimma University, Institute of Health Ethical Review Committee (reference number IHRPGD/553/2019). A support letter was obtained from the Health Research and Post Graduate director’s office for each study area. Written informed consent was obtained from participants over or equal to 18 years of age, while informed assent was obtained from study participants under the age of 18. Written informed consent were obtained from parents/guardians of participants below 18 years of age who visited the health facilities. All the participants' information was kept confidential using the coding system and no direct benefit was provided for the participants except cost-free lipid profile analysis and blood pressure checkup.

Five ml of venous blood collection was drawn by experienced nurses following the standard operating procedure for venous blood collection developed by WHO [24]. Those who were found to be positive for the measurements of this study were advised and linked to nearby health facilities for further evaluation. The used materials like syringe, gauzes, and leftover blood samples were disposed of according to the laboratory waste management protocol and incinerated.

## Results

### Behavioral characteristics

A total of 146 study participants (45 DMPA users, 51 Implant users, and 50 non-users) were included in the study. The result showed no significant mean age difference between DMPA users, implant users, and nonusers (*p* = 0.12). The mean age (years) of DMPA users, implant users, and non-users were 26.04 ± 4.76, 25.41 ± 4.2, and 24.28 ± 3.78 respectively.

The behavioral status of DMPA users, implant users, and non-users was matched. The results showed that all study participants in each group were eating an unhealthy diet, had no history of consumption of alcohol, and no habit of cigarette smoking. However, only 11% (n = 16) of the participants chewed khat once per week, but no participants reported chewing khat daily. Regarding physical activity, the ratio between physically active to physically inactive participants was almost 1:1 in each group*.*

### Anthropometric indices, blood pressure, and lipid profile status

Results from one-way ANOVA showed that there was statistically significant mean difference in Body Mass Index (BMI, *p* = 0.045), hip-to-waist ratio (*p* = 0.012), systolic blood pressure (SBP, *p* = 0.027), diastolic blood pressure (DBP, *p* = 0.017), TC (*p* = 0.005), LDL (*p* = 0.023) and TAG (*p* = 0.000) between the three groups of participants (DMPA users, implant users, and non-users) (Table 1).

**Table 1:** Comparisons of mean for (one-way ANOVA output) anthropometry, blood pressure, and lipid profile among the users and non-users in selected health facilities of Jimma Town, 2019

Parameters	Mean ± SD of the parameter			*p* value
	DMPA (n-45)	Implant (n-51)	Non-Users (n-50)	
*Anthropometric indices*				
Height in cm	158.1 ± 6.7	157.6 ± 8.9	161.2 ± 5.5	–
Weight in kg	54.1 ± 6.7	51.5 ± 7.2	52.6 ± 6.7	–
BMI	21.7 ± 2.6	20.7 ± 2.1	20.2 ± 2.5	0.045
Hip circumference in cm	95.5 ± 12.4	93.7 ± 9.7	94.6 ± 6.9	–
Waist circumference in cm	85.9 ± 9.4	81.2 ± 9.3	83.1 ± 6.7	–
Hip/waist	1.1 ± 0.1	1.16 ± 0.11	1.14 ± 0.05	0.012
*Blood pressure*				
SBP	113.3 ± 6.1	116.8 ± 6.5	113.9 ± 5.5	0.027
DBP	74.9 ± 6.7	79.4 ± 6.9	77.9 ± 9.2	0.017
*Lipid profiles*				
TC	158.7 ± 22.7	143.75 ± 23.6	144.5 ± 26.8	0.005
HDL	50.6 ± 8.8	50.9 ± 8.7	47.28 ± 8.7	0.071
LDL	91.2 ± 22.3	78.8 ± 18.2	83.6 ± 24.7	0.023
TAG	91.6 ± 20.9	66.7 ± 19.6	78.1 ± 23.9	0.000

#### Lipid profiles status of study participants

The Bonferroni post-hoc analysis of lipid profile revealed that the mean TC was statistically significantly higher among DMPA users compared to non-users (*p* = 0.016) and implant users (*p* = 0.01). DMPA users had a statistically significantly higher mean TAG than implant users (*p* = 0.000) and non-users (*p* = 0.008) (Table 2).

**Table 2:** Bonferroni post hoc output of lipid profile among each group of participants in selected health facilities of Jimma town, 2019

Dependent variable	(I) Type	(J) Type	Mean difference (I–J)	*p* value
TC	DMPA users	Implant users	14.96	0.010
		Non-users	14.23	0.016
	Implant users	DMPA users	− 14.96	0.010
		Non-users	− 0.73	1.000
HDL	DMPA users	Implant users	− 0.27	1.000
		Non-users	3.39	0.184
	Implant users	DMPA users	0.27	1.000
		Non-users	3.66	0.111
LDL	DMPA users	Implant users	12.39	0.019
		Non-users	7.642	0.273
	Implant users	DMPA users	− 12.34	0.019
		Non-users	− 4.75	0.829
TAG	DMPA users	Implant users	24.96	0.000
		Non-users	13.52	0.008
	Implant users	DMPA users	− 24.96	0.000
		Non-users	− 11.43	0.026

#### Blood pressure of the study participants

Implant users had significantly higher mean SBP compared to DMPA users (*p* = 0.038). There was a statistically insignificant lower mean SBP among DMPA users compared to non-users (*p* = 1.000). Implant users had higher mean SBP than non-users even though it was statistically insignificant (*p* = 0.107). DMPA users had significantly lower mean DBP than implant users (*p* = 0.015). There was a statistically insignificant lower mean DBP among implant users compared to non-users (*p* = 0.070) (Table 3).

**Table 3:** Bonferroni post hoc output of blood pressure among each group of participants in selected health facilities of Jimma town, 2019

Dependent variable	(I) Type	(J) Type	Mean Difference (I–J)	*p* value
SBP	DMPA users	Implant users	− 3.11	0.038
		Non-users	− 0.56	1.000
	Implant users	DMPA users	3.11	0.038
		Non-users	2.55	0.107
DBP	DMPA users	Implant users	− 4.51	0.015
		Non-users	− 2.99	0.183
	Implant users	DMPA users	4.51	0.015
		Non-users	1.51	0.979

#### Anthropometric indices of the study participants

Results from Bonferroni post-hoc analysis showed that DMPA users had significantly lower mean hip-to-waist ratio than implant users (*p* = 0.04). Implant users had higher mean hip-to-waist ratio (*p* = 0.9) while DMPA users had lower mean hip-to-waist ratio (*p* = 0.4) than non-users although in all cases, the differences were not statistically significant. DMPA users had a statistically significantly higher mean BMI than non-users (*p* = 0.01) (Table 4).

**Table 4:** Bonferroni post hoc output of anthropometric indices among each group of participants in selected health facilities of Jimma town, 2019

Dependent variable	(I) Type	(J) Type	Mean Difference (I–J)	*p* value
Hip/waist	DMPA users	Implant users	− 0.05	0.04
		Non-users	− 0.03	0.4
	Implant users	DMPA users	0.05	0.04
		Non-users	0.02	0.9
BMI	DMPA users	Implant users	1.02	0.12
		Non-users	1.45	0.01
	Implant users	DMPA users	− 1.02	0.12
		Non-users	0.43	1.00

### Correlation between duration of use and outcomes

**Fig. 1:**
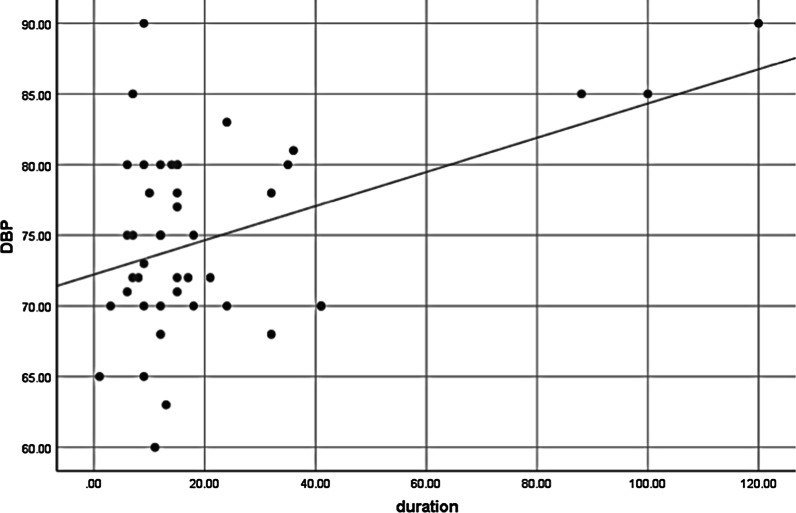
Correlation of DBP with the duration of DMPA uses among study participants in selected health facilities of Jimma town, southwest Ethiopia, 2019

The mean duration of DMPA and implant use was 21.82 and 15.27 months respectively. Results from bivariate Pearson correlation analysis showed that there was a statistically significant moderate positive linear relationship (a correlation of between 0.3 and 0.7) between the duration of DMPA use and DBP (*p* = 0.003 and r = 0.438) (Fig. 1) while other parameters (LDL, HDL, TAG, SBP) did not have significant linear relationship with duration of use (Table 5)*.*

**Table 5:** Bivariate Pearson correlation analysis for the association between duration of HC use in months with anthropometric indices, blood pressure, and lipid profiles among HC users in selected health facilities of Jimma town, 2019

	DMPA users	Implant users
*BMI*		
R	0.216	− 0.055
*p* value [2-tailed]	0.155	0.701
*Hip-to-waist ratio*		
R	-0.063	0.165
*p* value (2-tailed)	0.679	0.248
*SBP*		
R	0.072	− 0.176
*p* value (2-tailed)	0.637	0.218
*DBP*		
R	0.438	− 0.187
*p* value (2-tailed)	0.003*	0.190
*TC*		
r	0.296	0.077
*p* value (2-tailed)	0.048	0.589
*HDL*		
r	− 0.004	− 0.146
*p* value (2-tailed)	0.978	0.308
*LDL*		
r	0.122	0.238
*p* value (2-tailed)	0.425	0.093
*TAG*		
R	-0.081	0.058
*p* value (2-tailed)	0.597	0.685

## Discussion

### Anthropometric indices, blood pressure, and lipid profiles of DMPA users

Serum TC level is a major indicator of risk of coronary heart disease; for every 1% increase in the TC level, there is a 2% increase in the incidence of coronary heart disease [25]. The finding of this study showed that the mean TC was significantly higher among DMPA users compared to non-users*.* The finding is consistent with those of Fekade et al. [11], Huma et al. [26], and Bakery [27] which showed that DMPA users have significantly higher TC than non-users. This suggests that DMPA users may be at a higher risk of developing coronary heart diseases than non-users. However, other cross-sectional studies by Faddah et al*. *[10], and Bawah et al*.* [28], found nonsignificant changes in mean serum TC level in DMPA users compared to non-users. The difference may be explained by the age difference of the study participants. Participants in the study by Faddah and colleagues were aged between 25 and 30 years while those in the present study were between ages 17 and 35. Since our study included women approaching menopause, the sample may exhibit age-related elevated serum TC levels [29]. Variations in findings could also be due to differences in study settings and food habits. Most Ethiopians usually utilize imported edible palm oil containing a large proportion of saturated fatty acid that raises cholesterol and total cholesterol to HDL ratio [30].

In the present study, the mean TAG was significantly higher among DMPA users than non-users which is in line with the finding by Huma et al*. *[26] and Bakery [27]. Nevertheless, it is inconsistent with findings by Bawah et al*.* [28] and Fekadie et al. [11] which showed that there was a statistically insignificant difference in TAG among DMPA compared to non-users. The discrepancy may be explained by the difference in the study protocol. This study used comparative cross-sectional study design by matching behavioral characteristics that may affect the health outcomes of HC users and non-users like Khat chewing habits, cigarette smoking, physical activity, alcohol intake, and diet. In contrast, other studies used comparative cross-sectional design without matching the behavioral characteristics despite the significant influence of such factors on lipid metabolism in the body, which can mask the true effect of hormonal contraceptives on lipid metabolism [31–35]. The other possible reason for higher TAG among DMPA users than non-users may be due to androgenic activity associated with the method rather than activation of the progesterone receptors [36].

The results of this study showed that the mean BMI was significantly higher among DMPA users than non-users although differences in hip-to-waist ratio were not statistically significant. This finding is in line with that of a study by George et al*.* [21] which revealed that DMPA users have a significantly higher BMI than non-users. An animal study by Bakery and Abdullah also revealed that DMPA-treated rats have significantly increased weight gain than those not treated [27], although this is not consistent with the finding by Bawah et al. that showed statistically insignificant difference in body weight between DMPA users and non-users [28]. DMPA-associated weight gain may be due to its effects on the regulation of appetite and energy expenditure by reducing cerebrospinal fluid leptin levels or its glucocorticoid-like activity [37].

There was no statistically significant difference in both mean SBP (*p* = 1.00) and DBP (*p* = 0.183) between DMPA users and non-users in this study. The finding is consistent with that of a study by Bawah et al*.* [28], while it is not consistent with findings from a study in Ghana [21]. The studies found a significant difference in DBP and an insignificant difference in SBP among DMPA users and non-users.

Our study found that the duration of DMPA use had no statistically significant correlation with lipid profiles and anthropometric parameters. However, duration of using DMPA was positively associated with DBP but not SBP. This is consistent with findings by Fekade and colleagues [11] and Wahda and Al-Youzbaki [38]. In contrast, the study by Nagah et al. [39] showed that lipid profile indicators (TC, LDL, HDL and TAG) were positively associated with duration of use. This may be due to difference in the age of participants and study design. Participants in the study by Nagah and colleagues were aged between 25 and 35 years while those in this study were between 17 and 35 years old, with those aged 17–24 comprising 33.3% of the total participants. There is evidences that age is positively associated with changes in levels of serum lipid profiles TC and TAG among women aged ≤ 60 and LDL among women aged ≤ 70 years of age respectively [40] while women in perimenopausal period have higher TC, LDL and TAG compared to younger women [41].

### Anthropometric indices, blood pressure, and lipid profiles of implant users

The findings of this study show that the lipid profiles of implant users were not significantly different from those of non-users. However, implant users had significantly lower TAG than non-users, which is in line with the findings by George et al. [21]. A study by Bawah et al*.* [28] found significantly lower HDL cholesterol levels and TC among implant users compared to non-users, and no significant difference in TAG and LDL between the two groups. However, this study found no significant difference in TAG and LDL between implant users and non-users. A study in Switzerland found significantly lower plasma HDL and LDL among Implanon users compared to non-users [42]. Another study in Sudan found higher TC, TAG, and LDL among women using implants compared to non-users [39]. The variations in the findings on lipid profiles across studies may be due to differences in lifestyle, including diet, alcohol intake habit, and physical activity, as well as the type of implant that participants used (the Switzerland study included only Implanon while this study included both Implanon and Jadelle). Implanon contains 68 mg of crystalline etonogestrel with a daily release rate of approximately 30 μg/day [43], while Jaddle contains 75 mg of the progestin levonorgestrel with a daily releasing rate of 100 μg/day at month 1, followed by a decline to about 40 μg/day at 12 months and to about 30 μg/day at 24 months and beyond. Additionally, Etonogestrel is a third generation progestogen, while Levonorgestrel is a second-generation progestogen which has high androgenic effect than Etonogestrel [44]**.** These differences may affect lipid metabolism.

The findings of this study showed that the mean difference in BP between implant users and non-users was not statistically significant. Similarly, findings by Qifang et al*.* [45] and Bawah et al. [28] showed that neither systolic nor diastolic blood pressure was affected by the use of implants. In contrast, George et al. [21] found significantly higher DBP and lower SBP among implant users than non-users although differences in SBP were not statistically significant. Implant users had insignificantly lower mean BMI and hip-to-waist ratio compared to non-users, which is consistent with findings by Bawah et al. [28] and George et al*.* [21]. The duration of implant use was not significantly associated with lipid profiles, anthropometric measures, and blood pressure.

### Limitation of the study

One of the limitations of the study is its cross-sectional nature, which limits the ability to determine causal relationships between the use of hormonal contraception and the health outcomes considered. Additionally, the small sample size may limit the ability to detect statistically significant differences between users and non-users of hormonal contraception even when the differences are large. Moreover, the findings may not be generalized to all women using hormonal contraception in Ethiopia or other settings given that the study was conducted in purposely selected health facilities in one region of the country.

## Conclusion

The findings of this study showed that DMPA users had significantly higher serum levels for TC and TAG than non-users while implant users had significantly higher blood pressure than DMPA users. The findings of the study further show that implant users had lower lipid profiles, blood pressure and anthropometric indices than non-users. The findings suggest the need for family planning service providers to undertake appropriate client profiling before recommending a particular method to women seeking the services in order to minimize adverse health risks, especially for those who may have pre-existing conditions.

## Data Availability

The data used in this paper are available on reasonable request from the corresponding author.

## Abbreviations

ANOVA: Analysis of variant
APO: APO protein
BP: Blood pressure
CV: Cardiovascular
DBP: Diastolic blood pressure
DMPA: Depo-medroxyprogesterone acetate
HC: Hormonal contraceptives
HDL: High-density lipoprotein
LDL: Low-density lipoprotein
MPA: Medroxyprogesterone acetate
SBP: Systolic blood pressure
TAG: Triacylglycerol
TC: Total cholesterol
WHO: World Health Organization
